# Impact of spaceflight on gene expression in cultured human mesenchymal stem/stromal cells

**DOI:** 10.1371/journal.pone.0315285

**Published:** 2025-03-13

**Authors:** Peng Huang, Bryan T. Piatkowski, Yesesri Cherukuri, Yan W. Asmann, Abba C. Zubair

**Affiliations:** 1 Center for Regenerative Biotherapeutics and Department of Laboratory Medicine and Pathology, Jacksonville, Florida, United States of America; 2 Department of Quantitative Health Sciences, Rochester, Minnesota, United States of America; 3 Department of Quantitative Health Sciences, Jacksonville, Florida, United States of America; University of Hawai'i at Manoa, UNITED STATES OF AMERICA

## Abstract

With technological advancements, human’s desire to explore space is growing and more people are staying longer at the international space station (ISS). The impact of microgravity on stem cells (SC) is not fully understood. We explored the impact of microgravity on gene expression profile of cultured mesenchymal stem/stromal cells (MSCs) at the ISS. We also evaluated how the new knowledge gained sheds light on our understanding of human physiology on Earth. Primary cultures of MSCs were expanded at the ISS for 1 or 2 weeks and mRNA was isolated from samples of the cultured cells. Gene expression profiles were determined and compared with samples from real-time ground control cultures. Differential gene expression, gene set enrichment analysis and determination of key genes were performed that revealed for the first time the existence of potential “master regulators” coordinating a systemic response to microgravity. Cyclin D1 (CCND1), a protein-coding gene that regulates cell cycle progression and CDK kinases, was identified as the most connected regulator at week 1. Further analysis showed the impacted genes from cultured MSCs significantly correlated with known gene pathways associated with cell division, chromosomal segregation and nuclear division, extracellular matrix structure and organization, muscle apoptosis and differentiation. This study exemplifies the utility of space research to advance our understanding of human physiology both on Earth and in space.

## Introduction

Exploring other celestial bodies, such as Mars and the Moon, not only expands our knowledge of the solar system but also lays the foundation for future human colonization and the possibility of finding extraterrestrial life. Space exploration helps us understand the conditions necessary for supporting life and ensures the protection of Earth from potential threats, such as asteroid impacts [1]. In addition, Space missions contribute to advancements in human health research, including studies on bone loss, muscle atrophy [2], cardiovascular function [3], and the effects of radiation [4]. These findings have potential applications in improving healthcare and extending human longevity on Earth.

The impact of microgravity on SC is an area of active research, and while our understanding is still evolving, several effects have been observed. Microgravity has been shown to affect the differentiation potential of SC. Studies have reported changes in the ability of stem cells to differentiate into specific cell types, such as hematopoietic cell [5], and cardiomyocytes [6–8]. This altered differentiation potential may impact the regenerative capacity of SC in a microgravity environment.

Microgravity can influence the proliferation and viability of SC [9,10]. Some studies have shown that under microgravity conditions, SC exhibit reduced proliferation rates and increased cell death [11,12]. These effects may affect the overall population of SC and their ability to maintain and repair tissues.

Microgravity can lead to changes in the gene expression profiles of SC [13,14]. Studies have identified differential gene expression patterns in SC exposed to microgravity, indicating altered signaling pathways, regulatory factors, and cellular processes [6,15]. These changes may impact the behavior and functionality of SC [12]. The microgravity environment alters the physical forces and fluid dynamics that SC experience. This disruption can affect cell signaling, cell-cell interactions, and the biochemical cues that regulate stem cell behavior [16–18]. It may influence factors such as cell adhesion, cytoskeletal organization, and cell-matrix interactions, which are crucial for stem cell function. Microgravity has been shown to induce epigenetic changes in SC. These modifications involve alterations in DNA methylation patterns, histone modifications, and non-coding RNA expression [19,20]. Epigenetic changes can influence gene expression and cellular behavior, potentially affecting stem cell fate and function.

Understanding the impact of microgravity on SC is vital for future space exploration and the potential utilization of SC for regenerative medicine in space. It can also provide insights into the fundamental mechanisms governing stem cell biology and tissue homeostasis both on Earth and in space. True microgravity, as experienced on the International Space Station (ISS), offers the most precise and realistic environment for studying biological systems, providing invaluable long-term data. This comprehensive approach enables us to identify subtle changes and adaptations in MSCs that may not be apparent in short-term or simulated conditions. However, access to ISS is expensive and logistically challenging, with significant waiting periods for spaceflight opportunities. Conversely, simulated microgravity, created using devices like clinostats, is more accessible and cost-effective, allowing for frequent and flexible research, albeit with some limitations in replicating all aspects of true microgravity. Although simulated microgravity attempts to mimic the effects of true microgravity on human MSCs, it is impossible to completely achieve microgravity due to interactions with unavoidable factors like shear stress. The ISS environment also includes unique factors such as cosmic radiation and near-vacuum conditions, further distinguishing it from Earth-based simulations. Ongoing research aims to elucidate these effects and develop strategies to mitigate or harness them for therapeutic purposes.

Mesenchymal stem/stromal cells (MSCs) play a crucial role in human physiology and have diverse functions in various tissues and organs [21]. Due to their unique characteristics and functional versatility, MSCs hold great potential for regenerative medicine [22], tissue engineering [23], and therapeutic interventions for various diseases and injuries [24]. We have previously shown that MSCs maintain their phenotype and proliferative characteristics after expansion in microgravity, with no effect on their differentiation capacity into osteoblasts and adipocytes after two weeks. However, microgravity significantly altered the secretion of six cytokines and growth factors, including increased PDGF-AA and IFN-γ-inducible protein (IP-10) and decreased sCD40L, IL-10, MCP-3, SDF-1α/β after 2 weeks in microgravity environment relative to ground controls. These changes highlight the duration-dependent effects of microgravity on MSCs secretion profiles, enhancing their immunosuppressive capacity without compromising genomic integrity or inducing tumorigenic transformation [25]. Our study also indicates that microgravity has minimal impact on mesenchymal stem cells (MSCs) in terms of their ability to enter the S phase of the cell cycle within the first 7–14 days of culture. The expression of CDKN2A and E2F1 was not significantly affected by microgravity. However, Polo-like kinase 1 (PLK1) expression decreased significantly only after 14 days in culture aboard the ISS. While microgravity may slow MSC progression at later cell cycle stages during longer-term culture, it does not appear to reduce their overall growth rate compared to ground controls. Further studies are necessary to confirm these findings. Ongoing research aims to further understand and harness the capabilities of MSCs to advance medical treatments and improve human health. Overall, while our understanding of the role of MSCs in a microgravity environment is still limited, the available evidence suggests that microgravity can influence the behavior, differentiation potential, gene expression profiles, and paracrine signaling of MSCs [19,25]. Further research is needed to comprehensively explore the effects of microgravity on MSCs and to harness their potential in regenerative medicine and tissue engineering applications for space exploration and terrestrial healthcare.

We have used RNA sequencing (RNA-Seq) to quantitatively assess the impact of microgravity on gene expression profile of cultured human mesenchymal. RNA-Seq offers high sensitivity and provides a broad dynamic range for gene expression quantification. Gene expression profiling is valuable in studying developmental processes and stem cell biology. It can provide insights into the genes and regulatory networks involved in cell fate determination, tissue development, and organogenesis. Additionally, gene expression profiling of SC can help characterize their differentiation potential and guide their use in regenerative medicine.

## Methods

### Study design

**Fig 1:**
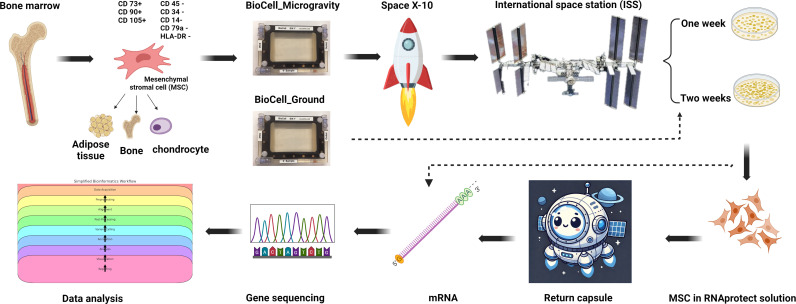
Overall study design. Scheme for generation of characterization MSCs, spaceflight experiment, sample collections, RNA extraction, sequencing, and gene expression analysis. Created in BioRender. Huang, P. (2024) https://BioRender.com/e45j781.

The experiments utilized BioServe’s single-well BioCell, a spaceflight-certified cell culture hardware capable of effectively supporting cell culture. MSCs-containing BioCells were transported to the ISS microgravity environment within Plate Habitats (PHABs). During launch rocket’s gravity forces can reach up to 5g. Upon arrival at the ISS, the PHABs were placed inside BioServe’s SABL unit, which maintains a temperature of 37°C and 5% CO2. Collected samples were frozen at temperatures below − 95°C once processed and remain frozen until their return to Earth. MSCs cultures were processed at two intervals: 7 days and 14 days. Seeding densities were adjusted such that no media exchange was performed at ISS. Ground control MSCs underwent identical treatment to those in the microgravity environment. Samples were preserved in RNAprotect (QIAGEN, Germantown, MD) or cryopreserved in cell preserving medium containing 5% dimethyl sulfoxide (DMSO) and returned to our laboratory for further analyses. Returned samples were characterized to establish their identity, purity, sterility, stability and functionality. The results of these analysis were separately published [25]. The overall study design is outlined in Fig 1. The study adhered to all ethical standards. The MSCs cell line was derived from an anonymized, healthy bone marrow donor. All procedures fully complied with relevant regulations and Mayo Clinic’s IRB guidelines.

### Cell culture

The MSCs were derived from a healthy, de-identified, discarded bone marrow sample using Histopaque-1077 (Sigma-Aldrich) according to density gradient protocols [25]. The MSCs were grown in α minimum essential medium supplemented with 16.5% fetal bovine serum. The expanded MSCs were then seeded into BioCells at passage 4. The BioCells were divided into groups for 1-week or 2-week durations. The 1-week group was seeded with 5x10^4^ MSCs, while the 2-week group was seeded with 2x10^4^ MSCs. Identical BioCells were prepared as ground controls.

### RNA-Seq and secondary analysis

At the designated termination timepoints, specifically after either one or two weeks of culture in space aboard the ISS, MSCs were subjected to trypsinization. This process facilitated the retrieval of single-cell suspensions, which were subsequently preserved in RNAprotect (QIAGEN, Germantown, MD) while still aboard the ISS. Upon return to Earth, RNA isolation was conducted utilizing the miRNeasy Mini kit (QIAGEN, Germantown, MD).

Total RNA concentration and quality were determined using Qubit fluorometry (Invitrogen, Carlsbad, CA) and the Agilent Bioanalyzer (Santa Clara, CA). cDNA libraries were prepared using up to 200 ng of total RNA according to the manufacturer’s instructions for the TruSeq RNA Library Prep Kit v2 (Illumina, San Diego, CA). The concentration and size distribution of the completed libraries were determined using the Bioanalyzer and Qubit fluorometry.

Libraries were sequenced at an average of 35 million, 100 base pair paired-end reads per sample with Illumina’s standard protocol for the Illumina cBot and HiSeq 3000/4000 PE Cluster Kit.

The FASTQ files were processed through the Mayo RNA-Seq bioinformatics pipeline, specifically using MAP-RSeq v3.0.2 [26]. This pipeline uses the STAR aligner v2.5.2b [27], which is both fast and accurate, and accounts for splice-awareness to create BAM files by aligning the sequencing reads to the human reference genome hg38. Subread v1.5.1 [28] was employed for gene and exon expression quantification, yielding raw gene counts. Quality control was performed using RSeQC v2.6.4 [29].

#### Differential gene expression analysis.

Gene expression profiles were compared between samples to identify differentially expressed genes. In the first set of analyses, we sought to identify those genes affected by microgravity (ISS vs. ground) within time points (weeks). An additional analysis was performed to identify those genes affected by culture duration (week 2 vs. week 1) while controlling for the effect of microgravity (ISS vs. ground). For each analysis, we first pre-filtered the data to keep only those genes with greater than 0.5 average transcript per million (TPM) across samples. Next, we identified differentially expressed genes using the raw counts in edgeR v4.0.3 [30]. As part of the edgeR analysis, raw counts were normalized using the trimmed median of mean of M values (TMM) method [31]. For the analyses performed to identify those genes affected by microgravity, sample pairing information was included as a covariate in the generalized linear model. For the analysis performed to identify those genes affected by culture duration, microgravity environment was included as a covariate, but sample pairing information was not as it was confounded with culture duration. Raw *p*-values were adjusted using the Benjamini-Hochberg procedure. Those genes with an adjusted *p*-value (p.adjust) <  0.05 and fold change greater than 1.5 (|log2FC|>0.585) were identified as differentially expressed.

#### Enrichment analysis and determination of key genes.

Pathway analysis of differentially expressed genes in response to microgravity was performed using Qiagen’s Ingenuity Pathway Analysis (IPA) software v107193442 (QIAGEN Inc., https://digitalinsights.qiagen.com/IPA). Briefly, Fisher’s exact tests were performed for each microgravity comparison to assess significant enrichment (p.adjust <  0.05) in IPA’s canonical pathways, biofunctions, and upstream regulators[32]. Gene set enrichment analysis (GSEA) was performed using GSEA v4.3.2 for the C2, C5, and Hallmark gene sets [33]. For GSEA analyses, the raw gene counts were first adjusted for the sample pairing covariate using ComBat-seq [25] within the sva v3.50.0 package in R, then converted to TPM, and finally pre-filtered as done for different gene expression analysis. To determine key genes (drivers) involved in the microgravity response, the overlap of genes between enriched pathways and gene sets from both IPA and GSEA was identified for each time point (week). We considered key genes to be those that had overlap across the majority of IPA and GSEA analyses. Using IPA, the total number of connections between key genes was predicted and the most highly connected gene was considered the central regulator [19]. Finally, we sought to determine which Gene Ontology (GO) Biological Process terms were over-represented by genes that were induced or repressed in each comparison using clusterProfiler v4.10.0 [34].

## Results and discussion

### Impact of microgravity on cultured MSCs

**Fig 2:**
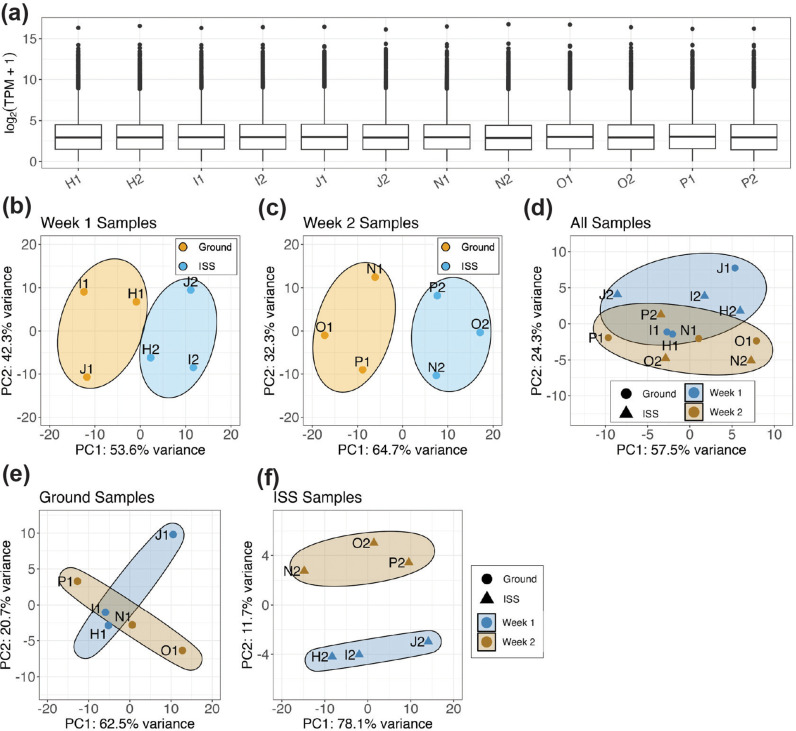
RNA-seq data are suitable to detect differential gene expression. (a) The overall distribution of normalized gene counts (transcripts per million, TPM) is similar for each sample. PCA score plots for samples collected at week 1 (b), week 2 (c), and both weeks (d). PCA score plots for samples collected from Ground controls (e) and ISS samples (f) were evaluated in relation to culture duration. In the PCA plots, normalized gene count data were first adjusted to account for sample pairing for comparisons within weeks and for microgravity environment for the comparison across time.

MSCs were cultured for 1 or 2 weeks aboard the ISS and this synchronized with real-time ground culture in our lab at the Mayo Clinic Florida. The overall distribution of normalized gene counts (transcripts per million, TPM) is similar for each sample. To visualize similarities among various MSCs samples, Principal Component Analysis (PCA) technique was utilized. PCA results show that MSCs samples had clear separation between the flight and ground controls (Fig 2a–d). In the comparisons between ISS and ground controls over time, we used ComBat-Seq to adjust gene counts for the sample pairing covariate. The separation is not clear when this pairing information is ignored. In the plot that includes all samples, sample pairing was confounded with the time period and could not be adjusted for. At this setting, the gene counts were adjusted based on location to enhance the visualization of the time effect. Therefore, the absence of separation in the plot is somewhat anticipated. When all MSCs samples were plotted separately based on location, there was clear differentiation between week 1 and week 2 among ISS samples but not in Ground controls (Fig 2e–f). The PCA graph shows strong clustering by location and duration, suggesting microgravity is the main driving factor for changes in gene expression overtime.

We also performed statistical test to identify genes that were differentially expressed between ISS and Ground location and culture duration for each given samples. We used an adjusted p-value (p < 0.05) cutoff and fold change as filtering criteria to reduce the probability of false positive genes (Table 1). Due to the variability in noise and the limited number of biological replicates (MSCs cultures were derived from single MSCs donor), we used 1-, 1.5-, 2-, 3-, and 4-fold change thresholds to produce a reasonable number of significant genes. High fold change led to a lower number of genes suggesting this approach was more stringent statistically and reduced chances of getting false positives. With this approach, we showed in 1-week cultures, the overall number of genes impacted were relatively small with a higher number of genes significantly repressed than induced at ISS compared to the ground control. While in the 2-week cultures there were about 20 times more genes impacted with no clear trend in number of significantly induced or repressed genes. Interestingly, comparing week 2 with week 1 cultures, there was significantly higher number of impacted genes with a clear trend towards induction of more gene expression in week 2.

**Table 1:** Summary of gene expression.

Comparison	Gene Filtering Criteria		Number of Genes		
	Adjusted *p*-value	Fold Change	Expressed	Induced	Repressed
ISS vs. Ground Week 1	None		22,511		
	0.05	1	52	19	33
		1.5	44	17	27
		2	15	4	11
		3	7	2	5
		4	4	2	2
ISS vs. Ground Week 2	None		22,647		
	0.05	1	1,043	550	493
		1.5	288	92	196
		2	78	31	47
		3	32	14	18
		4	23	10	13
Week 2 vs. Week 1	None		22,576		
	0.05	1	2,777	1,444	1,333
		1.5	735	404	331
		2	243	158	85
		3	87	57	30
		4	51	32	19

Location (ISS vs Ground) and time course (Week 2 vs Week 1) were selected to filter out differential gene expression profile. The higher fold changes the fewer genes were obtained in induced and repressed categories.

**Fig 3:**
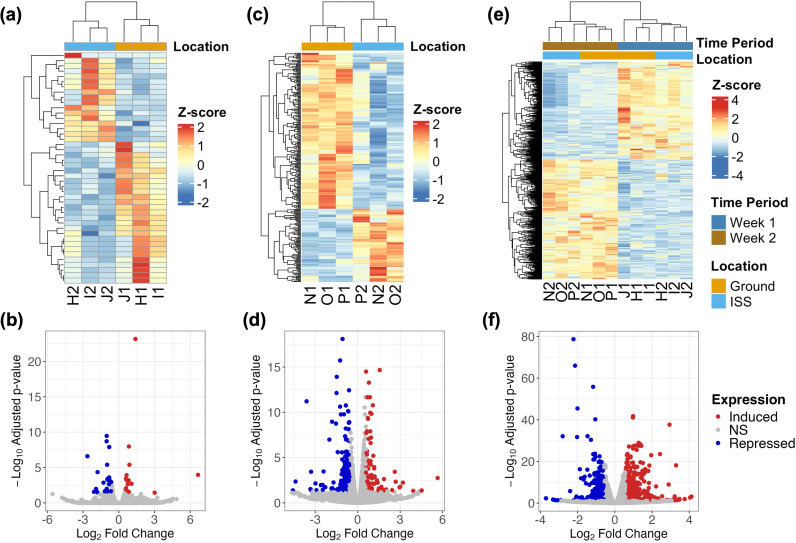
Differentially expressed genes (DEGs) were identified for each comparison. (**a**, **b)** Heatmap and volcano plots from the ISS vs. Ground comparison using Week 1 samples. Induced genes are those with higher expression in ISS samples. (**c**, **d**) Heatmap and volcano plots from the ISS vs. Ground comparison using Week 2 samples. Induced genes are those with higher expression in ISS samples. (**e, f**) Heatmap and volcano plots from the Week 2 vs. Week 1 comparison using all samples. Induced genes are those with higher expression in Week 2.

Furthermore, differentially expressed genes (DEGs) at the ISS relative to the Ground were identified for each comparison (Tables 1–2 and S1 Table) and visualized using heatmap and volcano plots representation with hierarchical clustering and Z-score values using Week 1, Week 2, and Week 2 vs Week 1 samples (Fig 3).

**Table 2.** List of key regulator genes at week 1.

Acronym	Full name	Function
CCND1	Cyclin D1	cell cycle progression
CUL1	Cullin 1	regulates cell proliferation, migration, and invasion
ID 1, 2, 3	Inhibitor of DNA binding 1, 2, 3	Cell proliferation and differentiation
PSMA2	Proteasome 20S subunit alpha 2	Proteasome function
PSMC4	Proteasome 26S subunit, ATPase 4	Proteasome function
PSMD14	Proteasome 26S subunit, non-ATPase 14	Proteasome function
RUVBL2	Human homologue of the bacterial RuvB gene	Homologous recombination and DNA double-strand break repair
THBD	Thrombomodulin	Binds thrombin
TNFRSF11B	TNF receptor superfamily member 11b	Bone remodeling

### Identification of key microgravity responsive genes and master regulators

**Fig 4:**
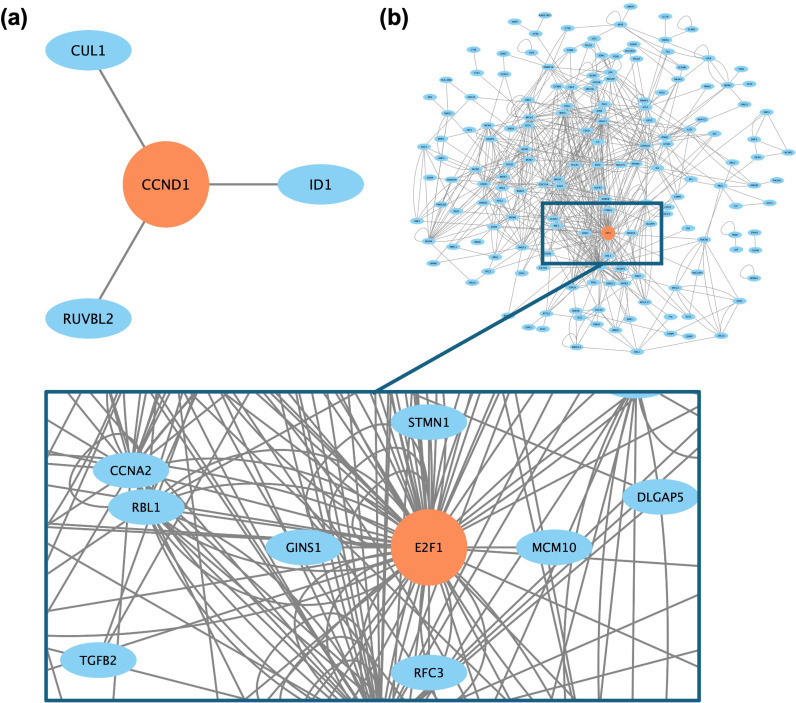
Key drivers for microgravity response at (a) Week 1 and (b) Week 2. For each comparison, key drivers were determined by identifying the overlap between those genes contributing to at least half of all significant IPA and GSEA enrichment analyses. Nodes represent genes and edges represent direct interactions in human as determined via IPA. Only those nodes with at least one edge are presented. The most highly connected node in each graph is shaded orange.

To determine key genes that impacted the physiological and biochemical processes in MSCs exposed to microgravity, we used a system biology approach as was previously reported [19]. In order to gain a comprehensive understanding of how microgravity affected MSCs, using Ingenuity Pathway Analysis (IPA QIAGEN Bioinformatics) and Gene Set Enrichment Analysis and the significantly differentially-expressed genes reported, we predicted key drivers of gene expression. To determine if any commonality exists between the sets of key genes for each dataset, we sketched connectivity within each dataset based on the number of biological connections occurring between key genes and determine which have the most influnece on observed gene expression (Fig 4). We hypothesize that genes within these biological pathways could be driving the transcriptomic response to spaceflight conditions. The specific connections between the key genes associated with each dataset can be considered as central hubs, such as those connections between CUL1, CCND1, ID1, and RUVBL2 within week 1 (Fig 4a). Key genes that were shared between the datasets were hypothesized to have the highest global impact on MSCs in response to microgravity. We identified CCDN1, CUL1, ID1, ID2, ID3, PSMA2, PSMC4, PSMD14, RUVBL2, THBD, TNFRSF11B as key genes at week 1 (Table 2), which have common pathways and have not previously been shown to be impacted by microgravity across various tissues except CCDN1 [35] and TNFRSF11B [36]. Furthermore, the ID1, ID2, and ID3 were also identified as key genes at week 2 (S1 Table). We identified the most connected key gene/driver to be CCND1 at week 1 and E2F1 at week 2.

### Evaluation of biological processes most impacted by microgravity responsive genes

**Fig 5:**
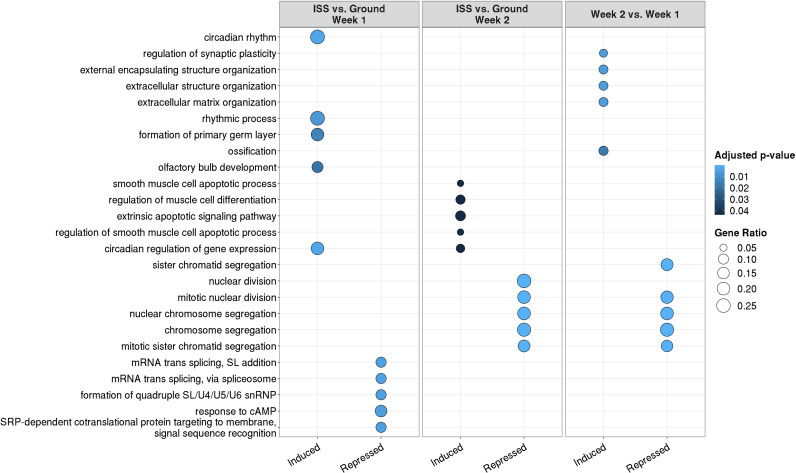
Top Gene Ontology (GO) enrichments for differentially expressed genes. For each comparison, the five GO Biological Process (BP) terms with the lowest adjusted p-value were selected from over-representation analyses of induced and repressed genes. Gene ratio refers to the proportion of differentially expressed genes in a given test that are annotated with that GO BP term.

Top Gene Ontology (GO) enrichments for differentially expressed genes are outlined in Fig 5. For each comparison, five GO Biological Process terms with the lowest adjusted p-value and their calculated gene ratios were selected from over-representation analyses of induced and repressed genes. Induced genes relative to ground control in MSCs following 1 week culture at the ISS appeared to correspond to genes associated with circadian rhythm while repressed genes corresponded with genes associated with mRNA splicing. However, with 2 weeks cultures, induced genes appeared to correspond with muscle cell apoptosis and differentiation associated genes while repressed genes corresponded with genes associated with chromosomal segregation and nuclear division. When we compared week 2 with week 1 cultures, genes associated with extracellular matrix structure and organization appeared to be induced in week 2 samples (Fig 5). We could not find any previous studies evaluating gene expression profile of human MSCs under true microgravity at ISS environment. Studies exploring the effects of simulated microgravity on human mesenchymal stem cells have shown that simulated microgravity affects the genomics, transcriptomics, epigenomics, metabolomics, and proteomics of hMSCs. These changes can alter cell structure, morphology, function, and dynamics, which are crucial for understanding how cells adapt to microgravity [37]. Simulated microgravity impacts bone-forming cells (osteoblasts) and hMSCs, which are essential for bone regeneration. This research is particularly relevant for mitigating bone loss in astronauts, as microgravity leads to significant bone density reduction [38]. Exposure to simulated microgravity can trigger epithelial-mesenchymal transition (EMT) in human keratinocytes, promoting migratory behavior and the expression of EMT transcription factors and markers. This transition is vital for understanding how cells change their phenotype in response to microgravity [39]. Simulated microgravity increases the proportion of certain hMSCs (CD226 + Lin−CD117 − Sca1+), which produce higher levels of cytokines like IL-6 and M-CSF. These changes can influence osteoclast differentiation and immune responses [40]. These studies highlight the complex biological responses of hMSCs to simulated microgravity, providing insights that are crucial for space medicine and potential therapeutic applications on Earth.

## Conclusions

The analyses presented here demonstrate the utility of the ISS environment to study the biological impact of microgravity. We found that, the ISS condition and culture duration generated several master regulators that resulted in major systemic responses towards microgravity. CCND1 is a cell cycle associated protein identified to be the most prevalent signaling node activated in response to microgravity along with 10 others being the most connected gene within week 1. CCND1 is a protein-coding gene that regulates cell cycle progression and CDK kinases [41]. It’s a key mediator of cell cycle progression and plays a vital role in the development and progression of cancer [42]. CCND1 is the main cyclin involved in the transition of cells from the G1 to S phase of the cell cycle [41]. It also regulates transcription factors, coactivators, and corepressors that control histone acetylation and chromatin remodeling proteins [43]. CCND1 also plays roles in cellular growth, metabolism, and differentiation [44,45].

The study’s limitations include using MSCs from a single donor, pre-seeding cells on Earth just before launch (exposing them to high gravity), and having a low seeding density that didn’t require media exchange during space culture.

Finally, our analyses suggest the global systemic response to microgravity in MSCs is driven by CCND1 and mediated by 11 key regulator genes at week 1. In conclusion, the current study demonstrates the value of the ISS environment in generating novel hypotheses through gene expression analysis. This study revealed microgravity-induced critical genes and signaling pathways that may be used to identify space related health risks and in the development of preventive measures that increase the safety of long-term manned missions. Future studies will involve using MSCs from multiple donors to further confirm our findings and allow for additional analysis of pathways responsive to microgravity conditions.

## Supporting Information

S1 Table Key genes identified from the overlap of IPA and GSEA enrichment analyses for weeks 1 and 2. (XLSX)
